# “Once is Enough” in Radiometric Calibrations

**DOI:** 10.6028/jres.112.003

**Published:** 2007-02-01

**Authors:** Gerald T. Fraser, Charles E. Gibson, Howard W. Yoon, Albert C. Parr

**Affiliations:** Optical Technology Division, National Institute of Standards and Technology, Gaithersburg, MD 20899-8440

**Keywords:** calibration, efficiency, process control, quality measurements

## Abstract

The successful development of an Optical Technology Division quality system for optical radiation measurement services has provided the opportunity to reconsider the existing calibration procedures to improve quality and reduce costs. We have instituted procedures in our calibration programs to eliminate uninformative repetitive measurements by concentrating our efforts on controlling and understanding the measurement process. The first program in our calibration services to undergo these revisions is described in this paper.

## 1. Introduction

The successful development of an Optical Technology Division quality system for optical radiation measurement services has provided the opportunity to reconsider the existing calibration procedures to improve quality and reduce costs. This effort leverages major advances in controlling manufacturing quality, led by W. E. Deming, in Japan, following World War II [1, 2]. These advances are equally applicable to the delivery of services such as calibrations. Deming and others developed approaches for controlling industrial processes to reduce product variability and, consequently, eliminate costly final product testing. For success, their approach requires manufacturers to understand and control all the steps in the production process so that variability in the final product is minimized to some desired tolerance level [3].

The early proponents of quality manufacturing developed statistical process control procedures and tests to help achieve low product failure rates. Their work has had a lasting impacting on efficiency and quality in product manufacturing. The control chart concept of W. A. Shewhart used for tracking some measurable output of a process is particularly relevant for the present discussion [4]. Shewhart stressed the understanding of the whole process and control of the variability in the elements of the process, to minimize inefficient final product testing. Deming made this point, as well, in his renowned 14 points of management, reproduced in Table 1. These points served as the foundation for the quality system that Deming developed. Points 3 and 5, in particular, are relevant to the improvement of the calibration process as they target the elimination of piecewise inspection, emphasizing, instead, control and improvement of the entire process.

Calibration services, like manufactured products, can be delivered with improved quality through effective control of the entire process. Such control eliminates the needless inefficiencies endemic in calibrations performed by government and industry scientists, whereby the entire measurement is typically repeated three or more times and averaged to obtain a reported result. Calibration scientists justify the repetition by claiming that the statistical uncertainty in the final result is lower, confidence is improved, and it has always be done this way. Such repeat measurements are, in part, a legacy of metrology’s early history when measurements where highly subjective, often involving a visual comparison. Details of this history for photometric and radiometric measurements will be given in Appendix 1. Here, we discuss the fallacy of maintaining such an approach and consider an alternative strategy whereby the calibration is performed only once. We denote this approach for convenience as “Once is Enough.”

To demonstrate the benefits of initiating a “Once is Enough” measurement strategy we consider one of the most popular optical radiation calibration services offered by the Optical Technology Division, the determination of the spectral irradiance of an appropriate lamp standard. Historically, the spectral irradiance of the test lamp has been measured three or more times over the entire 250 nm to 2400 nm wavelength region. The variation in the three measurements was used to assign a reproducibility uncertainty to the calibration, of unclear physical origin.

Despite extensive automation, repeat measurements are time consuming and needlessly tie up expensive instrumentation. They also incur approximately three times the wear on reference standard lamps, customer lamps, and motion controllers such as monochromator translation stages and scanning grating drives. Ultimately, calibration service customers pay for the extra cost associated with these additional measurements, without receiving any significant benefit. These customers tend to follow NIST’s lead and, likewise, perform repeat measurements with little benefit to “their” customers.

The repeating of a measurement is usually unnecessary for a well-characterized process, with a clearly understood and verified uncertainty budget for artifacts and a well characterized and understood stability and repeatability. It is only useful if it adds value to the state of knowledge of the measured quantity commensurate with the additional effort required. W. J. Youden, in his classic book [5], “Experimentation and Measurement,” comments on the repeating of measurements:

“Many people seem to feel that there is some magic in the repetition of measurements and that if a measurement is repeated frequently enough that the final result will approach a “true” value. This is what scientists mean by accuracy.

Suppose that you are in a science class and that the next two students to come into the room were a girl five feet ten inches tall and a boy five feet nine inches tall. Let each student in the class of 30 students already in the class measure the new arrivals to the nearest foot. The answer for both is six feet. Has the repeated measurements improved the accuracy?”

In Youden’s example, only one measurement is required to answer the problem poised. Repeat measurements are only necessary if they improve our knowledge of the measure and commensurate with the additional time and effort required. Over the last two decades, the spectral irradiance measurement service has improved significantly in performance, aided by advances in instrumentation, environmental control, and automation; by implementation of a quality system, and greater understanding of the intricacies of the measurement. The consequence of the improved performance is that the “reproducibility” component of the measurement is now small, eliminating the justification for repeat lamp measurements and providing the opportunity to implement “Once is Enough.” Below we discuss more fully the implementation of the “Once is Enough” strategy for spectral irradiance.

## 2. The Spectral Irradiance Calibration Process

**Fig. 1:**
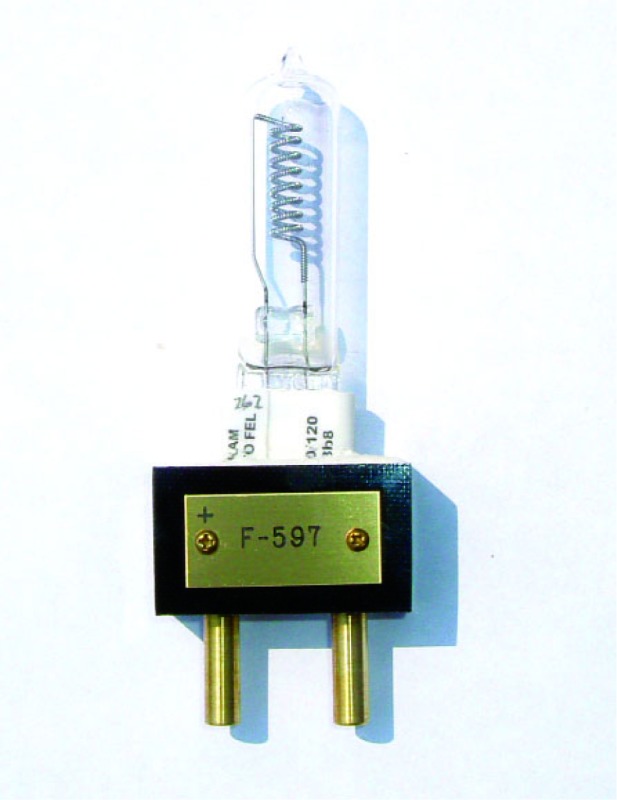
Photograph of a 1000 W quartz-halogen FEL lamp disseminated as a standard of spectral irradiance by NIST measurement services 39030C to 39046C.

**Fig. 2:**
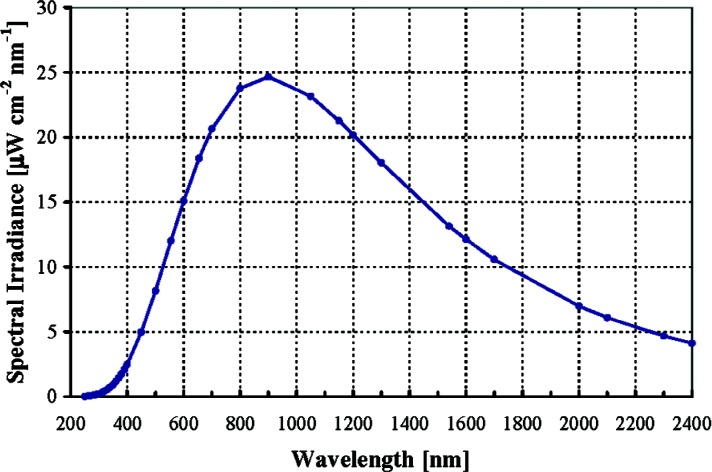
A typical spectral irradiance curve for an FEL lamp such as pictured in Fig. 1. The line is drawn to aid the reader. The spectral irradiance (optical power per unit surface area per unit spectral bandwidth) is plotted as a function of wavelength as measured 50 cm from the lamp.

The Spectral Irradiance Calibration Services 39030C to 39046C disseminate lamps with spectral irradiances measured as a function of wavelength. Typically, a 1000 W quartz-halogen FEL lamp is calibrated, where FEL is the lamp-type designation (not an acronym) of the American National Standards Institute (ANSI). A photograph of an FEL lamp is shown in Fig. 1 and a typical spectral irradiance curve, as measured 50 cm from a lamp, is shown in Fig. 2. The lamps are purchased from commercial sources and evaluated for suitability as standards [6]. The evaluation includes visual inspection of the bulb and filament structure, assessment of the spatial uniformity of the output, testing for the presence of strong atomic emission lines, and examination of the stability and relighting reproducibility. The lamps are also seasoned by operating at the prescribed current for 10 h to 15 h prior to cleaning and storing for future calibration.

The technical details of the calibration procedure and the associated measurement uncertainties are discussed in NIST Special Publication SP250-20 [6]. The calibration procedure and measurement uncertainties differ, somewhat, from that published initially some 20 years ago, and will be described in detail in an updated Special Publication [7].

**Fig. 3:**
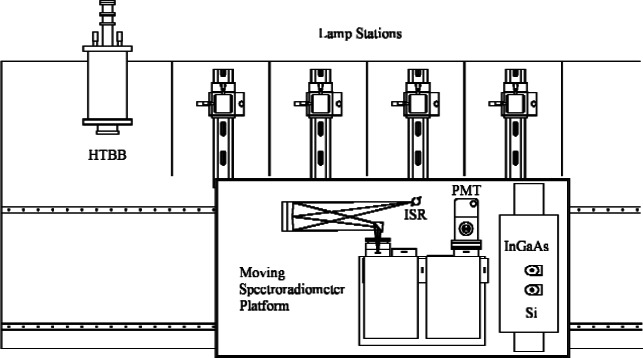
Schematic diagram of the second generation Facility for Automated Spectroradiometric Calibrations (FASCAL 2) with the scale realized from the high-temperature blackbody (HTBB) as collected by the integrating sphere receiver (ISR). For the ultra-violet wavelength regions, the photo-multiplier tube (PMT) is used instead of the Si or the InGaAs detectors.

Briefly, the spectral irradiance calibration compares the spectral output of a known lamp—the primary working standard (PWS)—to that of an unknown lamp, the lamp under test (LUT). Measurements are performed in the recently completed, second-generation Facility for Automated Spectroradiometric Calibrations, denoted as FASCAL 2, shown schematically in Fig. 3. The facility has four lamp stages, one for the PWS and three for the LUTs to be calibrated. The FASCAL 2 instrument consists additionally of a spectroradiometer, integrating sphere, and filter radiometer all mounted on a moveable carriage to align the entrance of the integrating sphere with either the PWS or with one of the three LUTs. The spectral irradiance of the PWS is calibrated by comparison with a blackbody, HTBB, whose radiance temperature is tied, by a set of filter radiometers, to the absolute cryogenic radiometer through the Spectral Comparator Facility (SCF) [8] or the Spectral Irradiance and Radiance responsivity Calibration with Uniform Sources (SIRCUS) Facility [9], as described by Yoon, Gibson and Barnes [10].

**Fig. 4:**
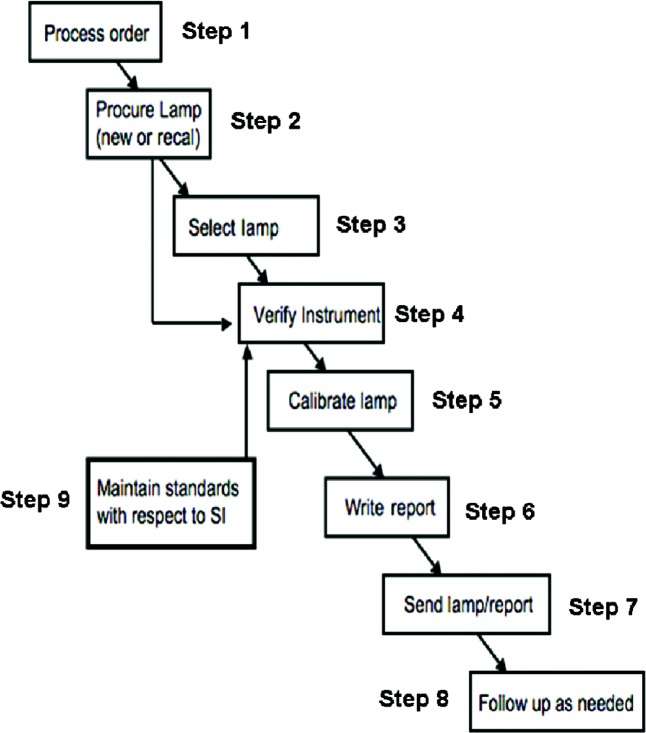
The nine steps required for the Spectral Irradiance Measurement Services. Steps 1 through 8 are routinely performed for each calibration. Step now is periodically performed to evaluate the irradiance scales maintained by the PWS lamps.

Delivery of the calibration service involves a number of steps, designated 1 through 9, as summarized in Fig. 4. The process starts with the acceptance by NIST of a request for calibration, and ends with the sending of the lamp and report to the customer, and addressing any follow-up issues, such as customer feedback, or specific lessons learned that can be applied towards improving the overall process. Such an analysis helps ensure continual improvement of the service and is a critical component of the associated quality system.

The core of the measurement process consists of steps 3, 4, 5, and supporting step 9, used to maintain the standards, with respect to the International System of Units (SI)[11]. These four steps consist of the physical measurements and the assignments of their measurement uncertainties. The development of a NIST calibration service entails the determination and validation of a complete uncertainty budget for the measurements. Validation is aided by international comparisons with other national metrology institutes. The uncertainty budgets are available to customers and other interested parties, as part of the public documentation for the service [6, 8].

## 3. Spectral Irradiance Calibration Uncertainty Budget

The present uncertainty budget for the spectral irradiance calibration service is summarized in Table 2. It varies slightly from that shown in Reference [10], but the differences are small and represent refinements in the determinations of specific components, rather than a substantial change in the overall measurement approach. We will briefly discuss these components to provide the context for the implementation of “Once is Enough.”

Lines 1 through 4 in Table 2 give the components contributing to the uncertainty in the radiance temperature of the High Temperature Black Body (HTBB) during calibration of the PWS lamps. The radiance temperature of the HTBB is determined using a set of filter radiometers with responsivities tied to the cryogenic radiometer. The HTBB is operated near 2950 K, to approximately match the spectral irradiance of the FEL lamp, thus reducing errors from spectral stray light and detector nonlinearity. The HTBB is well represented by a single radiance temperature, independent of wavelength, as determined by comparison against the small-aperture, NIST Variable-Temperature Blackbody (VTBB) [12]. The uncertainty associated with this conclusion is given in line 2 of the table, as “HTBB spectral emissivity.” When using the HTBB to calibrate the PWS, additional uncertainty components arise from spatial variation of the radiance temperature over the blackbody exit aperture (line 3), and from drift in the radiance temperature of the HTBB during the measurements (line 4).

Line 5, “geometric factors in irradiance transfer,” gives the uncertainty in the geometric factors required to define an irradiance level from the HTBB at the entrance aperture to the integrating sphere receiver of the spectroradiometer. The irradiance is given by

$$E_{\lambda ,\text{HTBB}}=L\left(\lambda ,T_{\text{radiance}}\right)\pi r_{\text{BB}}^{2}/D^{2},$$ (1)

where *L*(*λ*, *T*_radiance_) is the Planck radiance, *T*_radiance_ is the radiance temperature of the HTBB, *πr_BB_*^2^ is the area of the aperture in front of the blackbody, the modified distance factor is *D*^2^ = *d*^2^ + *r*^2^ + *r*_BB_^2^, where *πr*^2^ is the area of the entrance aperture to the integrating sphere receiver, and *d* is the separation between the two apertures. The uncertainty in *D* is dominated by the uncertainty in the separation between the two apertures, since *d* ≫ *r* and *d* ≫ *r*_BB_.

Lines 6 through 9 are the uncertainty components for the transfer of the blackbody-based irradiance scale to a PWS lamp by

$$E_{\lambda ,\text{PWS}}=f_{\lambda }\frac{S_{\lambda ,\text{PWS}}}{S_{\lambda ,\text{HTBB}}}E_{\lambda ,\text{HTBB}},$$ (2)

where *E_λ,_*_PWS_ is the irradiance of the PWS, *S_λ,_*_PWS_ and *S_λ,_*_HTBB_ are the spectroradiometer signals when measuring the PWS and HTBB, respectively, and *f_λ_* is the detector linearity correction factor associated with the different magnitudes of the signals measured when viewing the PWS and HTBB. Line 6, spectroradiometer responsivity stability, is the uncertainty component associated with the drift in the integrating-sphere/monochromator/detector system between measurement of the HTBB (*S_λ,_*_HTBB_) and the PWS (*S_λ,_*_PWS_). It is estimated using the standard deviation of the irradiance responsivities determined for three PWS lamps. Line 7 encompasses the effect of wavelength error in the monochromator drive on the spectral irradiance assigned to the PWS. The magnitude of this uncertainty component is strongly wavelength dependent, due to the shape of the spectral irradiance distribution of an FEL lamp. Line 8 contains contributions from uncertainties in *f_λ_*, stray light and spectral scattering. Line 9 is the uncertainty due to current instability in the lamp power supply. Note that line 8 and 9 terms are small and do not significantly affect the results. The terms in lines 1 through 9 are all type B uncertainties, uncorrelated with each other and with magnitudes assumed independent of the LUT being calibrated [13]. Since the PWS are measured against the HTBB only on a periodic basis, minimizing efforts in this aspect of the calibration process will only have minimal effects upon the total time spent in the calibration endeavor. The total uncertainty on the PWS irradiance calibration given in the table is derived from the root-mean-square sum of the individual uncertainty components.

The irradiance of the LUT, *E_λ,_*_LUT_, is determined by comparison against the PWS using

$$E_{\lambda ,\text{LUT}}=\frac{S_{\lambda ,\text{LUT}}}{S_{\lambda ,\text{PWS}}}E_{\lambda ,\text{PWS}},$$ (3)

where *S_λ,_*_LUT_ is the spectroradiometer signal when viewing the LUT. Note that *S_λ,_*_PWS_ in Eq. (2) is not identical to the *S_λ,_*_PWS_ in Eq. (3), since the measurements were performed at different times. Lines 10 and 11 are the uncertainty components associated with this irradiance transfer from PWS to LUT, which occur at the calibration of each LUT. Line 10 is the subject of this paper and is discussed in detail in the next section. Line 11 represents our experience on the stability of the irradiance scale of the primary working standard, as determined by repeat calibrations performed over several years, and intercomparison of PWS lamps [14].

Further work on the uncertainty determination will be carried out to formally take into account the correlations in the data. Correlations will come about because the individual wavelength determinations are correlated through the commonality of the reliance upon a common blackbody temperature. When these effects are fully considered the uncertainties could possibly lessen.

## 4. Lamp-to-Lamp Transfer

Initially, lamps disseminated to customers from the FASCAL facility were calibrated in groups of 12, by comparing their irradiances to that of four PWS lamps over the desired wavelength interval, typically 250 nm to 2400 nm [6]. Three LUTs and one PWS was mounted in the four FASCAL lamp stations for each of the 16 calibration runs required. According to the previous procedures, the LUTs and PWSs were permutated so that each LUT was calibrated four times, each time mounted in a different station and compared against a different PWS, also mounted in a different station. At each wavelength of the spectroradiometer the four lamps were measured and the wavelength was incremented. This process put about 6 h running time on each LUT.

The approach was intended to eliminate the possibility of disseminating a lamp calibrated against a PWS that had drifted out of calibration. It also attempted to reduce the effects of variations in the PWSs and in the lamp stations on the reported LUT irradiance values. At the same time it provided a set of four measured values for calculating a standard deviation for the repeatability of the calibration; a type A uncertainty component provided with the other uncertainty components in the customer calibration report.

The process was later modified in response to customer demands for faster and less expensive calibrations. Each LUT was now calibrated three times against three PWSs, instead of four times against four PWSs. As before, the LUT station, PWS, and PWS station were varied for each calibration of a LUT. Improvements in lamp temporal stability, through better lamp manufacturing and selection, reduced the magnitude of the PWS lamp drift during the measurements, providing additional opportunities to reduce the calibration time. This drift, which approximately and linearly depended on the number of hours the lamp had been operated since calibration, was effectively eliminated by changing the calibration procedure so the spectroradiometer scanned through an entire wavelength range once for each lamp, set by the responsivity range of the detector being used. Significant time was saved over the old process of viewing each lamp prior to changing the wavelength. The modified process continued with the launch of FASCAL 2.

**Fig. 5:**
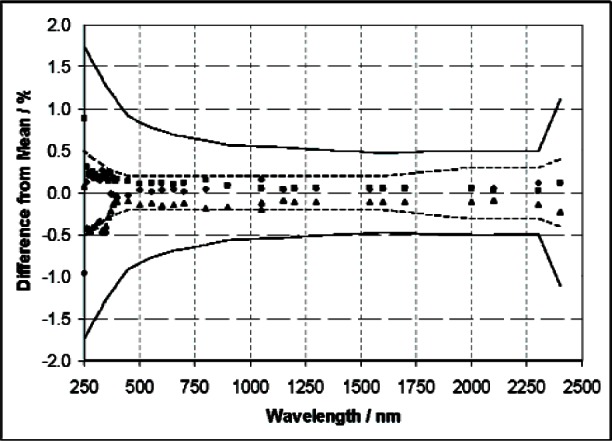
A plot showing the variation in the spectral irradiance assigned to an LUT positioned in stations 2, 3, and 4 of FASCAL 2. A single PWS mounted in station 1 was used for the measurements. Also shown in the figure are the total expanded (*k* = 2) uncertainties (solid lines), along with the expanded uncertainties for the lamp-to-lamp transfer (dashed lines) from line 10 in Table 2.

The requirement to have each LUT continue to be calibrated three times was driven by the desire to derive a standard deviation from the repeat measurements, to quantify PWS and station variability. The repeat calibrations also provided confidence in the calibration stability of the PWSs and equivalency of the lamp stations. Both of these rationales are insufficient for justifying the extensive amount of time, effort, and instrument and lamp hours expended on repeat calibrations, particularly given the level of agreement between different calibrations of the same lamp. As shown in line 10 of Table 2, the wavelength dependent lamp-to-lamp transfer uncertainty of 0.2 % to 0.5 % (*k* = 2) is small, relative to the total calibration uncertainty, which ranges from 0.49 % to 1.74 % (*k* = 2). This uncertainty component is based on a large number of lamp calibrations performed on FASCAL 2. Since there is a large sample set based on similar lamps to determine the repeatability uncertainty, two additional measurements do not add, in any significant way, to the state of knowledge about the calibration uncertainty. A typical example of lamp repeatability is shown in Fig. 5, for an LUT alternately positioned in three different stations (2, 3, and 4) and calibrated against the same PWS positioned in station 1. The variation in the lamp-to-lamp calibration is well within the lamp-to-lamp transfer uncertainty listed in Table 2. Also, the lamp-to-lamp repeatability is well inside the overall uncertainty of the measurement given in the last line of Table 2, and shown in Fig. 5. The low uncertainty on the lamp-to-lamp transfer provides the primary technical justification for implementing “Once is Enough.”

## 5. Implementation of “Once is Enough”

Here, we provide a strategy for successful implementation of “Once is Enough.” The four components described in detail below will ensure confidence by both calibration scientist and calibration service customers in the spectral irradiance values of lamps measured only once.

### Automation

Automation improves the repeatability of a measurement by eliminating subjective human factors associated with reading analog meters, aligning the spectroradiometer optical axis to the lamp position, setting the monochromator wavelength, fixing lamp current levels, and reading spectroradiometer output signals. As mentioned previously and discussed in Appendix 1, the large number of subjective readings and manipulations originally performed in radiometric measurements, and their associated potential for human error and ambiguity, played a major role in institutionalizing repeat measurements in the calibration services.

The present FASCAL 2 system is completely automated, with the only human intervention being the mounting and dismounting of the PWS and LUTs in their stations. The NIST-issued FEL lamps are mounted on brass, bi-post bases to be fixed in kinematic mounts, so lamp alignments are reproduced within the uncertainties without any adjustments. The axial positioning and distance setting of the lamp stations have been previously established and are not routinely adjusted with a change in LUT or PWS. Since a non-imaging integrating sphere is used as the collection source, the alignment is relatively insensitive to the off-axis tilt of the integrating sphere aperture, with respect to the LUT. After the lamps are mounted, the currents are turned up to their set points with computer control. After allowing time for the lamp outputs to stabilize, the spectroradiometer is automatically positioned to each station and swept through the selected wavelength range. The spectroradiometer output signals are recorded by the computer, converted to absolute spectral irradiances, and inserted into a text file for insertion into the calibration report template.

### Uncertainty budget

A rigorous uncertainty budget is critical for the implementation of “Once is Enough.” Such an uncertainty budget should be validated through independent measurements that are ideally based on both similar and different measurement methods performed by independent scientists. Uncertainty budgets in radiometry and photometry have conventionally included a “repeatability” component based on a simple statistical analysis of repeat measurements. As mentioned earlier in this article, this was the motivation for repeating the measurement on a particular lamp, up to three or four times, in the belief that the uncertainty could be decreased. This is somewhat misguided as Table 2, line 10, contains an estimate based on many measurements, of the sort, of uncertainty that pertain to the expected variation in lamp performance; and a few additional measurements do not improve the statistical quality at all.

The exact origin of the repeatability uncertainty component is often not clear, but certainly includes contributions attributed to other components in the overall uncertainty budget. The spectral irradiance “repeatability” component, denoted as lamp-to-lamp transfer uncertainty in Table 2, includes contributions from lamp-current drift and spectroradiometer responsivity stability, which were already accounted for in the previous lines in Table 2. The “long-term stability of the LUT” uncertainty component is not considered in Table 2, as this is the customers responsibility to estimate when they use the lamp. We note that such double counting unnecessarily increases the magnitude of the reported uncertainties on the calibrated irradiances.

To avoid double counting, a rigorous uncertainty analysis should be physics based, clearly tying each uncertainty component to the measurement equation. A physics-based uncertainty analysis provides a framework for developing a strategy to reduce the overall measurement uncertainty, and in particular, the components limiting measurement reproducibility. Without a true physics-based model, it is difficult to determine with a finite measurement set, whether the repeat measurements should be distributed normally about the true mean.

As discussed above, for the FASCAL 2 measurements, the “repeatability” lamp-to-lamp transfer uncertainty component is small, relative to the total uncertainty. This repeatability component conservatively reflects the repeatability of the measurements as shown in Fig. 5. Its small magnitude and likely over estimate—due to double counting—indicates that little is contributed to the knowledge of the spectral irradiance of the LUT by repeating the measurement three times; certainly not commensurating with the additional time or effort expended.

### Measurement process controls

Carefully selected measurement process controls and associated control charts help ensure correct instrument operation, scale stability, and final calibration accuracy. They further help the calibration scientist immediately identify problems arising in the measurement, eliminating the expenditure of time and resources to complete a calibration that will fall outside of acceptable quality levels. Process controls and control charts also eliminate unnecessary final product testing, typically performed through a repeat calibration, to ensure measurement quality. As discussed in the Introduction, Deming and Shewhart promulgated the elimination of final product testing and the integration of statistical process control in the manufacturing process, to ensure final product quality and performance.

For spectral irradiance measurements, two levels of process controls are being implemented. These process controls provide either a gross or fine level of assessment of instrument, PWS, and LUT performance throughout the calibration process. The process controls directly associated with the measurement of the spectral irradiances are being documented in a series of three control chart sets to help provide early warning of potential problems in the calibration process.

The first level of control occurs in the lamp selection process. A number of measurements are presently performed on a lamp prior to its acceptance into the calibration process. The spectral output of the lamp is examined to determine the presence and strength of atomic emission lines. These lines are noted in the calibration report. The lamp output is examined at 654.6 nm to ensure that it varies by less than 1 %, for a ± 1° angular displacement from the defined lamp axis, and that it drifts by less than 0.5 % over a 24 h period. The former measurement is performed to aid lamp users interested in realizing a spectral radiance scale by illumination of a highly reflective diffuser.

Three check standard (CS) lamps, calibrated against the HTBB, are used to assess the stability of the PWS lamps. The CSs are used infrequently to ensure the constancy of their spectral irradiance values. The PWSs, of which there are a total of six, are periodically used to assign a spectral irradiance scale to the CSs. The spectral irradiances determined for the CSs are required to stay within one FASCAL 2 standard uncertainty (i.e., half the *k* = 2 value from the last line of Table 2) of their initially calibrated value. The measurements are tracked in a set of control charts for easy referral; denoted here as Control Chart Set 1.

**Fig. 6:**
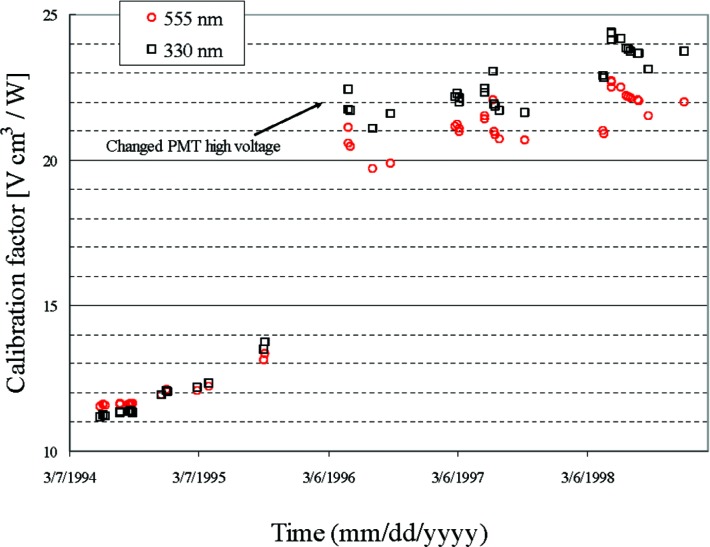
Plot showing the change in the spectral irradiance responsivity of FASCAL as a function of time at 330 nm and 555 nm. The nearly linear growth in responsivity with time is due to change in the sensitivity of the PMT used at that time. For FASCAL 2, a more stable silicon detector is used for wavelengths between 350 nm to 1050 nm, so that the drift in the spectral irradiance responsivity is dominated by drift in the reflectivity of the integrating sphere coating.

A second set of control charts, Control Chart Set 2, is being developed to track the spectral irradiance responsivity of the spectroradiometer as a function of time, defined as the ratio of the output voltage of the spectroradiometer to the input spectral irradiance from a PWS. Such control charts only provide evidence of gross change in the performance of the radiometer since, fractional changes in the responsivity over time are larger than the relative standard deviation of the reported spectral irradiances. An example of such a plot for two wavelengths is shown in Fig. 6, for FASCAL. The figure shows a nearly linear drift in responsivity with time, except for a sharp break, due to a change in the operating voltage applied to the photomultiplier tube. The drift is significant and is caused by a continual change in the photomultiplier tube sensitivity. Present silicon detectors used in FASCAL 2, between 350 nm and 1050 nm, have much less drift so that the change in spectral irradiance responsivity of the spectroradiometer within this wavelength interval is dominated by the change in the absolute reflectance of the integrating sphere coating; on the order of 0.5 %/day, for a newly coated sphere in the ultraviolet wavelength region to < 0.1 %/month for the visible and infrared wavelength region. Control charts will encompass one wavelength each in the ultraviolet, visible, and infrared to track the spectroradiometer performance, when used with the photomuliplier tube, silicon, and extended InGaAs detectors. The overall stability of the radiometer system is well understood and has a long recorded history that serves as an overall check on the system as significant deviations from the expected output of the system will be immediately recognized.

A third set of control charts (Control Chart Set 3) will be developed to track the ratio of the spectral irradiance of the PWSs as a function of time, again at the same three wavelengths. In the absence of a stable monitoring detector to assess the absolute output of a PWS, “Once is Enough” calibrations will be performed by comparing 2 LUTs against 2 PWSs mounted simultaneously in the four FASCAL 2 lamp stations. Sequential calibrations will position the PWSs, so all four stations are tested in the two calibration runs. The relative irradiances between PWSs should be predictable from the uncertainties in Table 2 if the instrument and the standards are performing as expected.

The assigned spectral irradiances of the LUT and the PWS can also be checked by filter radiometers calibrated for spectral irradiance responsivity. Since filter radiometers can have long-term stability of responsivity exceeding that of lamps, these filter radiometers can act as separate check of the assigned spectral irradiances. Work is being done to have stable filter radiometers calibrated for spectral irradiance responsivity to measure all FEL lamps put through the calibration process.

To perform calibrations in FASCAL 2 of three LUTs, against one PWS, requires that a monitoring filter radiometer be developed to track the absolute irradiance of the PWS at a selected wavelength. A silicon-detector-based filter radiometer will be used to obtain the necessary long-term stability. Ideally, the radiometer will operate in the ultraviolet where the lamp drift is greatest, to provide the most sensitive tracking of PWS stability. The challenge will be to find a detector-filter package whose long-term absolute responsivity in the ultraviolet is stable to better than 1 %.

Additional controls to track instrument performance include comparing newly assigned spectral irradiances, with previous measurements on a customer-submitted recalibration, comparing spectral irradiance scales with other standards laboratories through international comparisons and periodic scale realizations [14].

### Quality system

A quality system is critical for ensuring that a measurement is repeatable with the same uncertainty budget, independent of operator or time. A quality system, such as the ISO 17025 standard implemented at NIST, provides clear documentation of the measurement process to be followed by the calibration scientist. It also provides the foundation for implementing Deming’s points 3 and 5 in Table 1. The described measurement procedure should be identical to that validated through measurement intercomparisons.

### Peer review

Periodic objective technical peer review aids the calibration scientist in developing a technical rigorous and robust measurement approach and associated uncertainty analysis. It aids the elimination of aspects of the calibration not well founded in fundamental physics. For a mature calibration service, exploring, in detail, the origin of each component of the uncertainty analysis typically leads to the conclusion that measurement repeats are unnecessary.

Furthermore, the peer review often reveals steps in the measurement process that are done, more, by custom rather than for their contribution to the final result. Finally, an objective analysis can lead to useful suggestions to improve the overall calibration service. Such extensive peer review responds to the spirit of Deming’s points 1, 5, and 8.

## 6. Conclusions

“Once is Enough” is being implemented throughout the calibration programs within the Optical Technology Division. Calibration programs involved include optical properties of materials measurement, photometry, and radiometry. The detailed management and technical peer review undertaken as part of this effort has already led to significant improvements in the quality and reliability of the measurements. Such improvements include elimination of superfluous steps in the process, optimization of controls to ensure instrument performance, enhanced automation to maximize repeatability, and increased understanding of the uncertainty budget. We anticipate that “Once is Enough” will be fully implemented, division-wide, by the end of 2007. In addition to saving time, as much as 50 %, the increased focus on controlling and understanding the causes of variability in each step of the process will result in an improvement in the overall quality of our measurement services, for the benefit of NIST’s customers.

We anticipate that the lessons learned within the Division on the implementation of “Once is Enough” can be disseminated to other calibration services within NIST and to various calibration laboratories involved in supporting the U.S. National Measurement System. The concomitant reduction in time and costs will allow industry to reduce costs and improve measurement accuracy, to obtain an increased competitive advantage in areas such as manufacturing. Continued examination of best practices in manufacturing will, likewise, lead to new strategies to improve the performance and dissemination of NIST calibration services.

## Figures and Tables

**Table 1:** Deming’s 14 points of quality management

1.	Create constancy of purpose to improve product and service.
2.	Adopt new philosophy for new economic age by management learning responsibilities and taking leadership for change.
3.	Cease dependence on inspection to achieve quality; eliminate the need for mass inspection by building quality into the product.
4.	End awarding business on price; instead minimise total cost and move towards single suppliers for items.
5.	Improve constantly and forever the system of production and service to improve quality and productivity and to decrease costs.
6.	Institute training on the job.
7.	Institute leadership; supervision should be to help do a better job; overhaul supervision of management and production workers.
8.	Drive out fear so that all may work effectively for the organisation.
9.	Break down barriers between departments; research, design, sales and production must work together to foresee problems in production and use.
10.	Eliminate slogans, exhortations and numerical targets for the workforce, such as 'zero defects' or new productivity levels. Such exhortations are diversory as the bulk of the problems belong to the system and are beyond the power of the workforce.
11.	Eliminate quotas or work standards, and management by objectives or numerical goals; substitute leadership.
12.	Remove barriers that rob people of their right to pride of workmanship; hourly workers, management and engineering; eliminate annual or merit ratings and management by objective.
13.	Institute a vigorous education and self-improvement programme.
14.	Put everyone in the company to work to accomplish the transformation.

**Table 2:** Uncertainty Budget for Spectral Irradiance Calibrations Using FASCAL 2

Source of Uncertainty	Relative Expanded Uncertainties (k = 2) [%]									
	250 nm	350 nm	450 nm	555 nm	655 nm	900 nm	1600 nm	2000 nm	2300 nm	2400 nm
1. HTBB temperature uncertainty (0.86 K at 2950 K (B)	0.57	0.41	0.32	0.26	0.22	0.16	0.09	0.08	0.07	0.07
2. HTBB spectral emissivity (B)	0.10	0.10	0.10	0.10	0.10	0.10	0.10	0.10	0.10	0.10
3. HTBB spatial uniformity (B)	0.10	0.10	0.10	0.10	0.10	0.10	0.10	0.10	0.10	0.10
4. HTBB temporal stability (0.1 K / h) (B)	0.07	0.05	0.04	0.03	0.03	0.02	0.01	0.01	0.01	0.01
5. Geometric factors in irradiance transfer (B)	0.10	0.10	0.10	0.10	0.10	0.10	0.10	0.10	0.10	0.10
6. Spectroradiometer responsivity stability (B)	0.60	0.60	0.30	0.30	0.30	0.30	0.30	0.30	0.30	1.00
7. Wavelength accuracy (0.1 nm) (B)	0.58	0.26	0.13	0.07	0.04	0.01	0.01	0.01	0.01	0.01
8. Lamp/spectroradiometer transfer (B)	0.10	0.10	0.10	0.10	0.10	0.10	0.10	0.10	0.10	0.10
9. Lamp current stability (B)	0.07	0.05	0.04	0.03	0.03	0.02	0.02	0.01	0.01	0.01
**Total uncertainty of the primary working standards**	**1.03**	**0.80**	**0.50**	**0.45**	**0.43**	**0.40**	**0.37**	**0.37**	**0.37**	**1.02**
10. Lamp-to-lamp transfer (A)	0.50	0.30	0.20	0.20	0.20	0.20	0.20	0.30	0.30	0.40
11. Long-term stability of primary working standards (B)	1.31	0.94	0.73	0.59	0.50	0.36	0.20	0.16	0.14	0.14
**Overall uncertainty of the test with respect to SI units**	**1.74**	**1.27**	**0.91**	**0.77**	**0.69**	**0.57**	**0.47**	**0.50**	**0.49**	**1.11**

**Note:** The Type A or Type B evaluation of the uncertainty is indicated in parentheses.

## Appendix 1

NIST, and formerly NBS, has been performing radiometric and photometric calibrations since the founding of the Bureau of Standards in the early 20th century [15]. The early radiometric and photometric scales were based upon flame sources such as candles and gas lamps, later progressing to lamps and black-bodies. Measurements typically relied upon a visual comparison of a test source with a reference source [16]. The quantification and standardization of the relationship between human visual response and photometric and radiometric quantities [17, 18] allowed measurements of photometric quantities with detectors similar to those used in radiometry, but with appropriate filters to mimic the human visual response. In 1975, Blevin and Steiner proposed a redefinition of the candela from being based upon blackbody radiation to being based upon the amount of optical power at 555 nm, the peak wavelength for the human visual response [19]. The 1979 Conférence Générale de Poids et Mesures (CGPM) accepted the 1977 Comité International de Poids et Mesures (CIPM) recommendation to adopt this definition for the candela. This redefinition initiated a revolutionary change in the measurement of photometric quantities. Subjective measurements based upon visual inspection were replaced by objective measurements performed by electro-optical sensors. NIST eliminated such visual comparisons in their photometric and radiometric measurements and implemented additional step to improve radiometric and photometric calibrations [20], including the modernization and automation of the laboratory facilities and the establishment of an ISO Guide 25 compliant quality system [21, 22]. The quality system has since been updated to comply with the new NIST-wide standards based upon the newer ISO Guide 17025 [23].
